# Intestinal Microbial Composition of Children in a Randomized Controlled Trial of Probiotics to Treat Acute Gastroenteritis

**DOI:** 10.3389/fcimb.2022.883163

**Published:** 2022-06-14

**Authors:** Rachael G. Horne, Stephen B. Freedman, Kathene C. Johnson-Henry, Xiao-Li Pang, Bonita E. Lee, Ken J. Farion, Serge Gouin, Suzanne Schuh, Naveen Poonai, Katrina F. Hurley, Yaron Finkelstein, Jianling Xie, Sarah Williamson-Urquhart, Linda Chui, Laura Rossi, Michael G. Surette, Philip M. Sherman

**Affiliations:** ^1^ Cell Biology Program, Research Institute, Hospital for Sick Children, Toronto, ON, Canada; ^2^ Sections of Pediatric Emergency Medicine and Gastroenterology, Department of Pediatrics, Alberta Children’s Hospital, Alberta Children’s Hospital Research Institute, Cumming School of Medicine, University of Calgary, Calgary, AB, Canada; ^3^ Alberta Precision Laboratories – Public Health Laboratory (ProvLab), Department of Laboratory Medicine and Pathology, University of Alberta, Edmonton, AB, Canada; ^4^ Women and Children’s Research Institute, Stollery Children’s Hospital, University of Alberta, Edmonton, AB, Canada; ^5^ Children’s Hospital of Eastern Ontario, University of Ottawa, Ottawa, ON, Canada; ^6^ Departments of Emergency Medicine and Pediatrics, Centre Hospitalier Universitaire (CHU) Sainte-Justine, Université de Montréal, Montréal, QC, Canada; ^7^ Division of Emergency Medicine, Department of Paediatrics, Hospital for Sick Children, University of Toronto, Toronto, ON, Canada; ^8^ Division of Pediatric Emergency Medicine, London Children’s Hospital Health Science Centre, Department of Pediatrics, Western University, London, ON, Canada; ^9^ Pediatric Emergency Medicine, Izaak Walton Killam (IWK) Children’s Hospital, Dalhousie University, Halifax, NS, Canada; ^10^ Section of Pediatric Emergency Medicine, Department of Pediatrics, Alberta Children’s Hospital, Cumming School of Medicine, University of Calgary, Calgary, AB, Canada; ^11^ Department of Biochemistry and Biomedical Sciences, McMaster University Medical Centre, Hamilton, ON, Canada; ^12^ Division of Gastroenterology, Hepatology and Nutrition, Department of Paediatrics, Hospital for Sick Children, University of Toronto, Toronto, ON, Canada

**Keywords:** bacteria, children, gastroenteritis, intestine, lactobacillus, microbiome, probiotics

## Abstract

Compositional analysis of the intestinal microbiome in pre-schoolers is understudied. Effects of probiotics on the gut microbiota were evaluated in children under 4-years-old presenting to an emergency department with acute gastroenteritis. Included were 70 study participants (n=32 placebo, n=38 probiotics) with stool specimens at baseline (day 0), day 5, and after a washout period (day 28). Microbiota composition and deduced functions were profiled using 16S ribosomal RNA sequencing and predictive metagenomics, respectively. Probiotics were detected at day 5 of administration but otherwise had no discernable effects, whereas detection of bacterial infection (P<0.001) and participant age (P<0.001) had the largest effects on microbiota composition, microbial diversity, and deduced bacterial functions. Participants under 1 year had lower bacterial diversity than older aged pre-schoolers; compositional changes of individual bacterial taxa were associated with maturation of the gut microbiota. Advances in age were associated with differences in gut microbiota composition and deduced microbial functions, which have the potential to impact health later in life.

**Clinical Trial Registration:**

www.ClinicalTrials.gov, identifier: NCT01853124.

## Introduction

Unless there is an insult to health, such as either an intercurrent gastrointestinal infection or exposure to antibiotics, the composition and function of the intestinal microbiome in adults is relatively stable over time (Chen et al., 2021). By contrast, during the first few years of life, the diversity of the bacterial composition of the gut microbiota increases with increasing age. Multiple factors influence microbial colonization of mucosal surfaces following birth and during infancy. Such factors include the perinatal environment (Selma-Royo et al., 2020), mode of delivery (Mitchell et al., 2020), duration of exclusive human milk feedings (Savage et al., 2018), exposure to antibiotics (Shao et al., 2019), and intercurrent acute intestinal infections (Chen et al., 2017). Perturbation of the infant microbiota has been linked to the later development of asthma and atopic dermatitis (Tun et al., 2021; Vu et al., 2021), which often persists into adulthood (Baron et al., 2020).

Several studies have explored the use of probiotics as a sole or adjunctive treatment for varying gastrointestinal distresses in the pediatric population, with varying degrees of clinical success (Perceval et al., 2019). However, many of these studies focus solely on clinical outcomes and occasionally perform microbial profiling after treatment to compare the effect of probiotic administration to a randomized control group (Forster et al., 2021). This approach, while standard, often overlooks the gut microbiota heterogeneity of pediatric populations (Roswall et al., 2021), and precludes investigators from evaluating individual responses to probiotic treatment. Understanding the effect of probiotic administration within participants and stratified by participant characteristics empowers investigators to determine underlying mechanisms and explore how probiotic administration may impact both gut microbiota composition and functions of these microorganisms.

Analysis of the microbiome within the pediatric population highlights the importance of understanding how age influences microbial population dynamics in health (Roswall et al., 2021) and disease (Dinleyici et al., 2018). Investigating disease-microbiome-functional pathways-chronological age and temporal associations in the pediatric population will provide a broader understanding of the dynamics of the microbiome during both health and disease.

We took advantage of the availability of stool samples collected from children from 3 months to 48 months of age who were enrolled into a prospective randomized trial that compared the severity of an acute gastroenteritis episode between those who received a probiotic mixture, containing *L. rhamnosus* and *L. helveticus*, to those who received a placebo (Freedman et al., 2018; Freedman et al., 2021b).

In this study, sequencing of the V3 and V4 regions of 16S rDNA was used to determine the bacterial composition and Picrust2 employed to then deduce bacterial functions in stool samples obtained at the time of acute gastroenteritis, after 5 days of receiving either the probiotic mixture or placebo, and after recovery from the acute illness at 28 days after entry into the study. Herein, in analysis of the primary outcome of the study we provide evidence that a five-day exposure to probiotics results in transient colonization of the gut, but does not induce detectable persisting changes in either the fecal microbiota composition or deduced bacterial functions. Rather, analyses of data for secondary outcomes showed that chronological age and evidence of an acute, intercurrent bacterial infection are determinants of bacterial diversity in the intestine of children under 4 years of age.

## Materials and Methods

### Study Design

This sub-study was performed as part of the investigator-initiated, multi-center, Probiotic Regimen for Outpatient Gastroenteritis Utility of Treatment (PROGUT) randomized, double-blinded, placebo-controlled trial (Freedman et al., 2018). In this study, children under four-years-old presenting to an emergency department with acute diarrhea received either a five-day course of a combination probiotic composed of lyophilized powder containing 4.0×10^9^ colony-forming units of two bacterial strains - *Lacticaseibacillus rhamnosus* (formerly *Lactobacillus rhamnosus* (Zheng et al., 2020) strain R0011 and *Lactobacillus helveticus*, strain R0052 - in a 95:5 ratio, or an identical-appearing placebo (Freedman et al., 2018). The contents of one sachet of probiotics or placebo, maintained at a temperature between 0° and 25°C, was sprinkled into 30 ml of the child’s preferred liquid twice daily.

### Illness Assessment

Severity of the acute illness was assessed by using the Modified Veskari Scale score (Schnadower et al., 2013; Freedman et al., 2018; Freedman et al., 2021a).

### Stool Collection, Detection of Known Enteric Pathogens and Measurement of fecal sIgA

As previously described (Freedman et al., 2018), stool samples were collected in the emergency department prior to discharge home. If a sample was not provided prior to discharge, the caregiver was provided with a stool collection container and instructions on specimen collection at home. A study-funded courier was used to retrieve stool samples within 12 hours of collection.

Pathogen detection was performed using the Luminex xTag Gastrointestinal Pathogen panel performed by Alberta Provincial Laboratory for Public Health, as previously described (Freedman et al., 2020).

As part of an *a priori* planned sub-study, levels of secretory IgA in stools were measured using a commercial immunoassay, as previously described (Freedman et al., 2021a).

### DNA Extraction and 16S Ribosomal RNA Gene Sequencing

To evaluate the effects of probiotic administration on gut microbiota composition, V3-V4 16S rRNA gene sequencing was performed on stool specimens collected from participants at baseline (day 0), 5 days after administration of either probiotics or placebo administration (day 5), and after a washout period (corresponding to day 28 of the study). Sequences of the 16S rRNA gene variable 3–4 (V3–V4) regions were amplified using modifications previously described (Whelan et al., 2014) and sequenced using the Illumina MiSeq platform (San Diego, California). Primer and adaptor sequences were trimmed from the resulting sequences using Cutadapt (Martin, 2011). DADA2 pipeline was used to filter and trim paired reads (Callahan et al., 2016) DADA2 error correction was performed for each paired read, the de-noised reads merged, and any sequences identified as chimeric sequences were removed.

Taxonomy was assigned to the resulting Amplicon Sequence Variants (ASV) using RDP classifier trained with the Silva v123 16S rRNA database (Quast et al., 2013). ASVs not assigned to bacteria were removed, and alpha and beta diversity analyses were performed on a rarefied ASV table using Phyloseq and Vegan packages in R v3.5.6. The predicted metagenomic function of the gut microbiota composition was performed using Phylogenetic Investigation of Communities by Reconstruction of Unobserved States (Picrust2) (Douglas et al., 2020).

### Statistical Analyses

Analysis of 16S rRNA data was performed in R v3.5.6. Alpha diversity and beta diversity were assessed using a rarified amplicon sequence table at 20,000 reads. Linear regression and repeated measure PERMANOVA (adonis2) were used to assess statistical significance between groups for alpha and beta diversity, respectively. Pairwise comparisons were assessed using the emmeans package in R and multiple comparisons were corrected with Tukey’s *post-hoc* adjustments. Differential relative abundance between treatment groups was assessed using a two-sided permutation t-test. Multiple comparisons were corrected using false discovery rate (FDR). Linear discriminant analysis (LefSE) (Segata et al., 2011) was used to compare microbiota composition between placebo and probiotic treated study groups after 5 days of intervention.

Probiotic species monitoring was performed by extracting all ASV with taxonomy assigned to *Lactobacillus*. *Lactobacillus* ASV counts were normalized by centre log ratio transformation and linear regression analysis was performed using Lmer package in R to identify *Lactobacillus* ASV associated with treatment; pairwise comparisons were performed using emmeans R package. ASV that were identified as significantly associated with treatment group and differential between probiotic and placebo-treated on day 5 were evaluated for strain level taxonomy using the complete ASV sequence compared to the 16S rRNA sequences for *L. rhamnosus* R0011 (GenBank: AGKC00000000.1) and *L. helevticus* R0052 (NC_018528.1) using BLASTn alignment (Altschul and Gish, 1996).

A mixed model linear regression analysis on centre log transformed genus level ASV was employed to identify taxa associated with both participant age, as a categorical variable defined as infants under one year of age (<1.0 yr), toddlers between one to two years of age (1.0 – 2.0 yrs) and pre-schoolers over 2 years of age (>2.0 - <4.0 yrs), and sampling day (days 0, 5 and 28 after entry into the study). Multiple comparisons were corrected using false discovery rate (FDR). The average centre log ratio normalized abundance was taken for each of the significant taxa across groups at the individual sampling time points (days 0, 5 and 28) and hierarchical clustering using complete linkage was employed to identify groups of taxa with similar abundances.

The core microbiota was determined by evaluating taxa with > 50% prevalence within each age group at both day 0 and day 28. Predicted microbial functional pathway abundance was normalized using centre log ratio. The mixed model linear regression analysis test with false discovery rate (FDR) used for multiple comparison correction was employed to assess changes in pathway abundance across both days in the trial and age groups.

Principal coordinate analysis was performed using total sum scaled predicted functional pathways abundance in STAMP (Parks et al., 2014). Differential abundance between age groups was determined by one-sided Welch’s t-test, with multiple comparisons corrected by FDR. Spearman correlation between predicted microbial pathways and participant age, measured in months after delivery, was performed using a centre log ratio transformed ASV extracted from study day 28 samples, and then corrected for multiple comparisons using FDR.

## Results

### Descriptive Statistics of Study Samples

All participants who had adequate residual stool samples collected at both day 0 (study entry) and 5 days post randomization (day 5) after an *a priori* planned analysis of fecal secretory immunoglobulin A (sIgA) levels were included in the current stool microbiota analysis, day 28 samples were included if adequate sample was available. Seventy participants (n=32 in the placebo group; n=38 in the probiotic arm of the trial) of the 133 PROGUT participants were included in this study. The clinical characteristics of participants included in the present study are displayed in **Supplemental Table 1**. A total of 208 stool samples were analyzed: two specimens failed to amplify by polymerase chain reaction, and, therefore, were not included, resulting in a total of 206 individual stool samples (69 from day 0, 69 five-days after entry into the study, and 70 samples at day 28, recovery period) from 70 participants that were analyzed in this report (**Supplemental Table 1**).

### Intestinal Colonization With Probiotic Species

**Figure 1:**
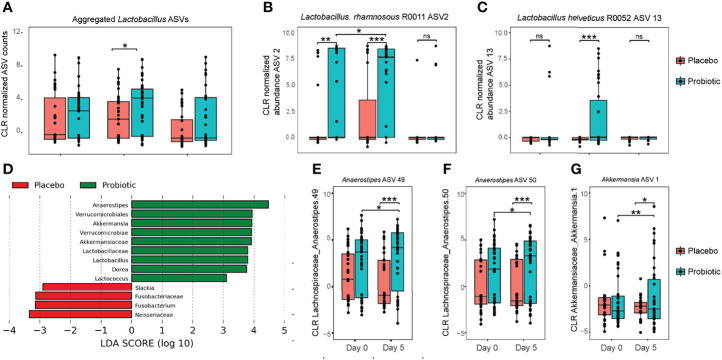
Genus level bacteria taxa during treatment with probiotics. **(A)** Center log ratio normalized abundance of ASV aggregated to Lactobacillus at the genus level; **(B)** center log ratio normalized abundance for Lactobacillus ASV2; and **(C)** centre log ratio normalized abundance for Lactobacillus ASV13. **(D)** Linear discriminant analysis effect size (LEfSe) analysis identified the most differentially abundant taxa between probiotic and placebo measured at study day 5; **(E**–**G**) using linear mix model regression, center log ratios with normalized abundance of individual amplicon sequence variants (ASV) were significantly associated with both probiotic intervention and changes across time. Levels of Akkermansia increased between study day 0 and day 5 in participants randomly assigned to the probiotic treatment study group. Significance was assessed using linear mixed modeling, pairwise comparisons were performed using estimated marginal means, with Tukey’s multiple comparison testing. Significance is denoted as *P < 0.05, **P < 0.01, ***P < 0.001 versus placebo; NS denotes not statistically significant.

To detect probiotic species *L. rhamnosus* R0011 and *L. helveticus* R0052 in either the probiotic- or placebo-treated participants, 16S rDNA sequences with assigned taxonomy of *Lactobacillus* were evaluated for significant changes in abundance between treatment groups and over sampling time points. Abundance of aggregated *Lactobacillus* ASVs were significantly increased in the probiotic treated group after 5 days of administration (**Figure 1A** **;** P=0.04). At an individual sequence level, 4 *Lactobacillus* ASV were identified as significantly changing with both probiotic treatment and sampling day.

To investigate strain level identity of Lactobacillus species, the two ASV sequences were compared for sequence homology against 16S rRNA sequences for probiotic species *L. rhamnosus* R0011 and *L. heleveticus* R0052 respectively: ASV 2 had 100% identity and 100% coverage with *L. rhamnosus* R0011, and ASV 13 an identity of 99.77% and only 1 gap to *L. helveticus* (**Supplemental Appendix**). Both ASVs were significantly increased in abundance in the probiotic-treated group only on day 5; abundance was reduced to the same as what was observed at baseline (study day 0) at day 28 (**Figures 1B, C**), indicating transient gut colonization of the two probiotic strains. Notably, ASV2, representing *L. rhamnosus*, was increased in relative abundance at baseline (day 0) between the probiotic and placebo study groups (p<0.01). Despite this baseline difference an increase in ASV2 abundance was also observed in participants who entered into the probiotic treatment group between baseline and day 5, which was not observed in the participants randomized to receive placebo.

### Effects of Probiotics on Bacterial Abundance at the Genus Level

Inference about relative abundance differences between probiotic- and placebo-treated participants after 5 days of intervention was investigated using linear discriminant effect size (LEfSe): a total of 13 bacterial taxa were identified as differential between the two study groups. At the genus level, *Anaerostipes, Akkermansia, Lactobacillus, Dorea* and *Lactococcus* were each enriched in the probiotic treated group, whereas *Slakia* and *Fusobacterium* were enriched in the placebo group (**Figures 1D–G**). To account for baseline differences between participants and the repeat measures design, a mixed model analysis was performed on the top 50 amplicon sequence variants (ASV) across Day 0 and Day 5, with probiotic treatment and sampling time points set as fixed factors and participant identification as random. Only 3 ASV were found with a significant interaction between probiotic treatment and sampling day: two *Anaerostipes* and one *Akkermansia* ASV were increased in abundance with probiotic treatment.

#### Probiotic (*L. rhamnosus*, Strain R0011 and *L. helveticus* R0052) Treatment for 5 Days Does Not Alter the Composition of the Gut Microbiota

**Figure 2:**
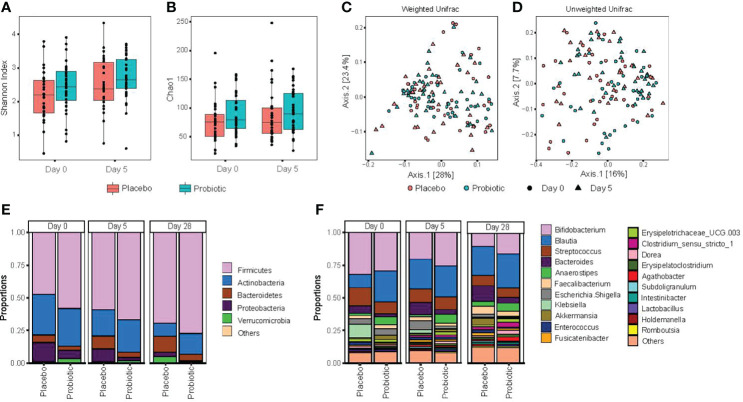
Probiotic treatment does not significantly alter gut microbiota composition or diversity. Stool samples were collected at the time of acute enteritis and entry into the study (day 0), 5 days after either probiotic or placebo (day 5) and 28 days following entry into the study (day 28). **(A)** alpha diversity, as measured by Shannon index; **(B)** alpha diversity species richness metric Chao1; **(C)** principal coordinate analysis of beta diversity, measured by Weighted Unifrac distances; **(D)** principal coordinate analysis assessed by unweighted Unifrac distances. Statistical significance was assessed using repeated measure (PERMANOVA); **(E)** relative abundance of gut bacteria characterized at the phylum level; and **(F)** relative abundance of the top 20 genus level taxa. Significance was assessed using two-sided permutation t-test, with multiple comparisons corrected by false discovery rate (FDR).

For analysis of the primary outcome of this study, individual species richness and diversity was measured using alpha diversity measures of both Shannon index and Chao1 **(** **Figures 2A, B**), but there was no statistically significant difference between placebo- and probiotic-treated participants after 5 days of treatment. However, despite randomization of participants, these differences were also present at baseline (day 0) between study groups; therefore, it is not clear if the differences observed after probiotic treatment represent a true effect of the intervention with probiotics.

Significant increases in alpha diversity were identified within participants over time (**Figures 2A, B**): with an increase in alpha diversity observed among both placebo- and probiotic-treated participants at day 28 (during recovery from acute enteritis) relative to study entry (day 0, with symptoms of acute gastroenteritis). Overall, no compositional differences, as measured by phylogenetic beta diversity metric weighted and unweighted Unifrac (**Figures 2C, D**), were found between probiotic and placebo treatment when accounting for baseline measurements (repeated measure PERMANOVA p > 0.05). Comparing relative abundance at the phylum level between probiotic- and placebo-treated participants at day 5 revealed significant decrease in *Proteobacteria* and *Bacteroidetes* among children entered into the probiotic arm of the randomized clinical trial (**Figure 2E**). Comparing genus level relative abundance after 5 days of probiotic/placebo treatment revealed increases in *Bacteroides, Anaerostipes* and *Dorea* and lower abundance of both *Klebsiella* and *Holdemanella* in children administered the probiotic; however, these differences were also evident between probiotic and placebo randomized participants at baseline (day 0; **Figure 2F**).

#### Baseline Differences in the Gut Microbiota is a Key Factor in Determining the Effects of Probiotics

**Figure 3:**
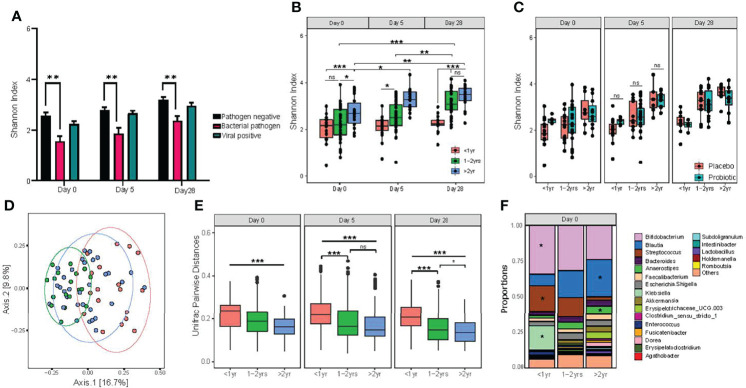
Detection of bacterial enteric pathogens and participant age both impact on the diversity of gut microbiota composition. **(A)** differences in alpha diversity associated with pathogen carriage status; **(B)** alpha diversity of age categories, as measured by the Shannon diversity index; **(C)** alpha diversity, measured by Shannon index, between probiotic and placebo treat groups within age categories. Statistical significance was assessed by two-way ANOVA, with Tukey’s multiple comparison testing; **(D)** principal coordinate analysis of unweighted Unifrac distances. Significance was assessed by using PERMANOVA; **(E)** pairwise dissimilarity distance comparison between age categories on study day 0, day 5 and day 28 using unweighted Unifrac distances. One-way ANOVA followed by Tukey’s multiple comparison testing; and **(F)** relative abundance of the top 20 genus level taxa, with statistically significant differences between age groups for *Bifidobacterium*, *Blautia*, *Streptococcus*, *Anaereostipes* and *Klebsiella*. Significance denoted by *P < 0.05, **P < 0.01 and ***P < 0.001. NS indicates no statistical significance.

To investigate secondary outcome variables in this study, a linear regression analysis within subjects was employed to evaluate the effects of patient characteristics on bacterial alpha diversity in the intestine, including the detection of enteric pathogens, baseline disease severity was measured using the Modified Veskari Scale score, antibiotic use in the previous fourteen days before study entry and the chronologic age of study participants. Both bacterial enteropathogen detection (P =0.0002) and participant age (P <0.001) were associated with the alpha diversity of the fecal microbiota (**Figure 3A**). Participants with positive bacterial enteropathogenic detection had a lower alpha diversity at baseline (study day 0; P<0.001). Alpha diversity of participants having a bacterial enteric pathogen detected increased slightly by day 28 (P=0.01) and remained lower compared to those with non-bacterial infections (P <0.001). Among the 11 participants with bacterial pathogens detected in their stool, 8 had *Clostridioides difficile* (5 subjects < 1 year of age), 2 were infected with *Salmonella*, and the remaining participant was positive for *Escherichia coli* O157:H7. *C. difficile* detection itself was associated with decreased alpha diversity of the gut microbiota at baseline (day 0; P= 0.034) and at all three time points throughout the study (P= 0.0002).

### Chronological Age Is a Dominant Factor in Determining the Overall Diversity of the Gut Microbiota

As shown in **Figure 3B**, the age of the participant was the most dominant significant factor affecting alpha diversity of the gut microbiome. For the purposes of this study, age was categorized into three distinct categories: <1.0 yr), 1.0 – 2.0 years, and >2.0 - <4 years). An effect of participant age on fecal alpha diversity was found at entry into the study (day 0; P=0.006), with participants >2.0 - <4.0 years exhibiting the highest microbial diversity. The largest difference between age categories was found between participants >2.0 - <4.0 years of age and those <1 year (P=0.005), although the difference between those >2.0 - <4.0 years and 1.0 - 2.0 years of age was also statistically significant (P=0.04).

As measured by using the Shannon index, alpha diversity increased over the 28-day study period for participants over 1 year of age. By contrast, participants under 1 year of age had no detectable increase in alpha diversity across the study period. To investigate the effect of age in response to probiotic treatment, we evaluated alpha diversity between participants who received the probiotic versus placebo within the stratified age categories of <1.0 year, 1.0 - 2.0 years of age, and >2.0 - <4.0 years old. There were no significant differences detected between the three age groups (**Figure 3C**).

Evaluating the effects of clinical and individual participant characteristics on beta diversity of the gut microbiota, participant age was also a dominant factor in determining differential gut microbiota composition at baseline (P=0.006; **Figure 3D**). By contrast, no significant effects related to the presence of enteric pathogens (either bacterial or viruses) were detected. Pairwise comparisons of Weighted Unifrac distances revealed that the largest difference was between infants <1.0 year and children >2.0 - <4.0 years; however, all pairwise comparisons were significant at baseline (day 0). Similar to alpha diversity, differences in beta diversity among age groups were also identified at both days 5 and 28 (**Figure 3E**). We then evaluated differences in relative abundance for taxa at the genus level among the three age categories at day 0 and found significant differential abundances for *Bifidobacterium, Streptococcus* and *Klebsiella*, which all significantly decreased with age, while *Blautia* and *Anareostipes* were increased (**Figure 3F**).

### Effects of Chronological Age on Gut Microbiota Composition at the Genus Level

**Figure 4:**
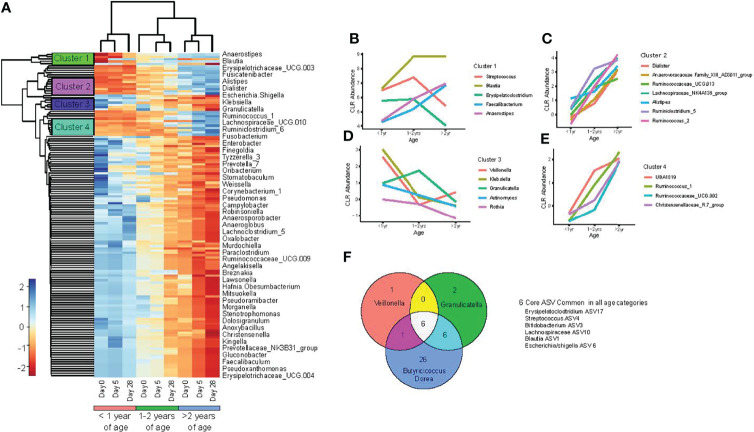
Changes in the gut microbiota occur with increasing chronological age. **(A)** heat map of bacterial taxa, with significant interactions between age of study participants and sampling day, as determined by linear mixed model regression; **(B–E)** mean center log normalized abundances of specific bacteria; and **(F)** Venn diagram comparison of core ASV between age categories (<1.0 year, 1.0 -2.0 years, and >2.0 - <4.0 years old), determined at study entry (day 0).

To further consider how participant age impacted gut microbiota composition over the study period and to account for individual differences, we performed a mixed model linear regression analysis on centre log transformed genus level ASV. This allowed the identification of bacterial taxa associated with both participant age as a categorical variable and sampling time points (days 0, 5 and 28). The average centre log normalized abundance was taken for each of the significant taxa across age groups at individual sampling time points and hierarchical clustering then used to identify groups of taxa with similar abundance. A total of 76 taxa were found significantly associated with age category and sampling time points (**Figure 4A**).

To further characterize trends in bacterial abundance and age under healthy conditions, we plotted the abundance of taxa within the individual hierarchical clusters at day 28 among the three age categories. One cluster consisted of *Streptococcus, Blautia, Erysipeloclostridium, Faecalibacterium Anaerostipes*, where *Blautia, Faecalibacterium* and *Anaerostipes* exhibited a linear relationship of increased abundance associated with age (**Figure 4B**). Two additional bacterial clusters exhibited all taxa identified to increase linearly with age, which were dominated by members of the *Ruminococcus* and *Ruminoclostridum* genus (**Figures 4C, E**). Lastly, one of the 4 identified clusters was composed of opportunistic pathogens: *Veillonella, Klebsiella, Actinomyces*, and *Granulicatella*, which were all found to decrease with advancing age (**Figure 4D**).

### Effects of Chronological Age on the Core Gut Microbiome

To understand how the core fecal microbiota composition differed between children of varying ages, we compared core ASVs for each age group at baseline (day 0) and during recovery from acute gastroenteritis (study day 28). Among all three age categories, six core ASVs were shared, including: *Veillonella, Erysipelatoclostridium, Streptococcus, Bifidobacterium, Blautia, and Escherichia/Shigella.* At baseline, infants <1.0 year had the smallest and least diverse core microbiome composition, with a total of 8 ASV identified consisting of 6 genera: *Veillonella, Erysipelatoclostridium, Streptococcus, Bifidobacterium, Blautia*, and *Escherichia/Shigella* (**Figure 4F**). Toddlers 1.0 – 2.0 years old had a slightly larger core microbiota, with 14 ASV, organized into 9 genera, including all those observed in infants <1 year, plus *Collinsella, Granulcatella Anareostipes* and*, Faecalibacterium*. Pre-schoolers >2.0 - <4.0 years old had the largest and most diverse core microbiota, with 39 ASV organized into 21 genera. The core gut microbiota for each of the three age groups remained the same at day 28 of the study compared with study entry at day 0, indicating that there was no appreciable effect of acute gastroenteritis and no effect after treatment with either probiotic or placebo on the core microbiota composition.

### Predicted Microbial Functions Associated With Chronological Age

**Figure 5:**
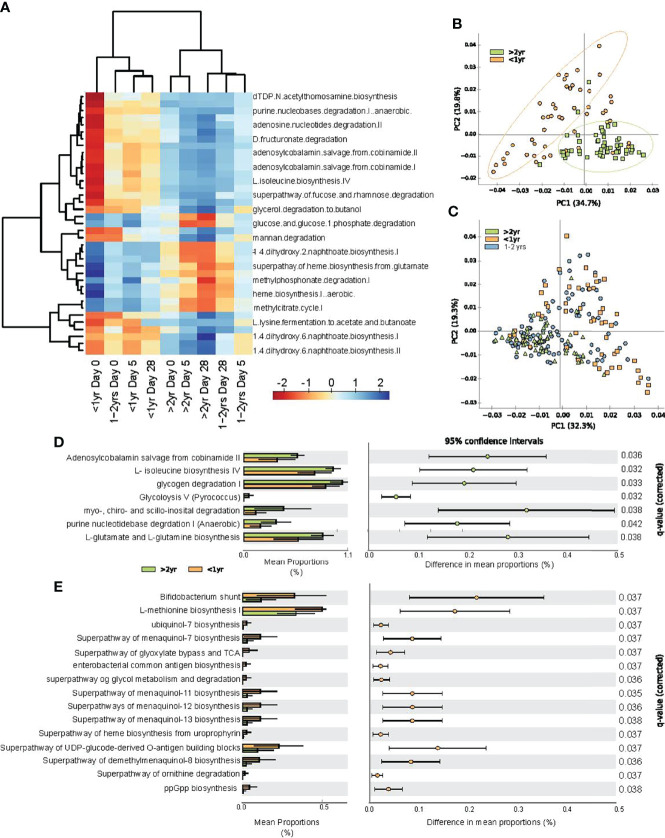
Predicted functional changes in gut bacteria across study periods and between age categories. **(A)** heat map of center log ratio normalized abundance of functional pathways were significantly associated with participant age category and sampling day, as determined by linear mixed model regression; **(B)** principal component analysis of predicted functional pathways at all sampling time points for study participants <1.0 year and those >2.0 - <4.0 years of age; **(C)** principal component analysis of all subjects at all time points; **(D)** extended error plot of abundance of functional pathways greater in children >2.0 - <4.0 years old compared to those <1.0 year; and **(E)** extended error plot of abundance of functional pathways, which were increase in infants <1.0 year. Statistical significance was assessed by one-sided Welch’s t-test, with multiple comparisons corrected by FDR.

To characterize deduced functional impacts of differences in the gut microbiota, we next performed predictive metagenomics using Picrust2. Mixed model linear regression analysis was used to identify differentially abundant pathways among the three age categories and across sampling time points, while accounting for repeat measures and individual participant variance. A total of 37 pathways differed among age groups and sampling time points (**Figure 5A**). Hierarchical clustering of the differentially expressed pathways revealed two distinct clusters. Participants <1.0 year at all sampling time points clustered together with subjects 1.0 – 2.0 years old at baseline (day 0).

Principal component analysis revealed clustering of predicted microbial functions for participants <1.0 year and over >2.0 – 4.0 years old, at every time point throughout the study (**Figure 5B**). Participants 1.0 - 2.0 years old exhibited functional pathways that were transitioning from the immature state to that of the >2.0 -<4.0 old (**Figure 5C**). To evaluate deduced microbial functional differences associated with age under healthy (recovered) conditions, we compared the relative abundance of specific pathways between participants <1.0 year and those >2.0 - < 4.0 years at study day 28. Participants >2.0 - <4.0 years old had increased vitamin B12 salvage and increased amino acid biosynthesis, as characterized by increases in isoleucine, glutamate and glutamine biosynthesis pathways (**Figure 5D**), whereas those <1.0 year were characterized by increases in menaquinol (vitamin K2) biosynthesis and heme biosynthesis (**Figure 5E**).

### Correlations Between Deduced Functional Pathways and Chronological Age

We further performed correlations between predicted metagenomic pathway abundance and participant age, expressed in months, at study day 28 (**Table 1**). Three pathways involved in biosynthesis of heme negatively correlated with age: super pathway of heme b biosynthesis from glutamate (*ρ* = −0.45), heme biosynthesis I aerobic (*ρ* = −0.47), and super pathway of heme biosynthesis from uroporphyrinogen III (*ρ* = −0.43). Furthermore, there was a negative correlation with several menaquinol and dimethyl-menaquinone biosynthesis pathways with increases in participant age. We also detected a positive correlation with 1,4 dihydroxy-6-napththoate biosynthesis, part of the separate futalosine pathway (Hiratsuka et al., 2009), and increasing age (*ρ* = 0.51). This pathway has been linked with anaerobiasis (Zhi et al., 2014), indicating that a shift in the production of menaquinone *via* differentially abundant pathways could be correlated to the transition to a more strictly anaerobic gut microbiota composition with increasing age.

**Table 1:** Correlation between predicted metagenomic pathways abundance at day 28 of the study and chronological age in months.

Pathways	(ρ) Rho^1^	Adjusted P value^2^
Super pathway of dimethyl menaquinol 6 biosynthesis I	-0.55	1.9E-04
Super pathway of dimethyl menaquinol 9 biosynthesis	-0.55	1.9E-04
Super pathway of menaquinol 6 biosynthesis I	-0.52	3.6E-04
Super pathway of menaquinol 10 biosynthesis	-0.52	3.6E-04
Super pathway of menaquinol 9 biosynthesis	-0.52	3.6E-04
Super pathway of glyoxylate bypass and TCA	-0.48	1.4E-03
TCA cycle IV 2-oxoglutarate decarboxylase	-0.47	1.4E-03
Super pathway of glycolysis pyruvate dehydrogenase TCA and glyoxylate bypass	-0.47	1.5E-03
Glyoxylate cycle	-0.47	1.7E-03
Heme biosynthesis I aerobic	-0.46	2.0E-03
Super pathway of heme biosynthesis from glutamate	-0.45	2.8E-03
Super pathway of methylglyoxal degradation	-0.43	3.9E-03
4-hydroxyphenylacetate degradation	-0.43	3.9E-03
Enterobactin biosynthesis	-0.43	3.9E-03
Super pathway of heme biosynthesis from uroporphyrinogen III	-0.43	4.2E-03
L-arginine degradation II AST pathway	-0.42	5.1E-03
Polymyxin resistance	-0.42	5.1E-03
PpGpp biosynthesis	-0.41	5.1E-03
Creatinine degradation II	0.34	2.4E-02
Gluconeogenesis I	0.35	2.3E-02
Super pathway of polyamine biosynthesis II	0.35	2.2E-02
Purine nucleobases degradation I anaerobic	0.35	2.2E-02
Adenosine nucleotides degradation II	0.36	1.8E-02
Arginine ornithine and proline interconversion	0.36	1.5E-02
Octane oxidation	0.38	9.9E-03
Super pathway of UDP N acetylglucosamine derived O antigen building blocks biosynthesis	0.41	5.4E-03
Isopropanol biosynthesis	0.42	5.1E-03
UDP 2,3 diacetamido 2,3 dideoxy alpha D mannuronate biosynthesis	0.42	5.1E-03
Urea cycle	0.43	4.2E-03
Mannan degradation	0.43	3.9E-03
Acetyl CoA fermentation to butanoate II	0.44	3.5E-03
Super pathway of menaquinol 8 biosynthesis II	0.45	2.9E-03
1,4 dihydroxy-6 naphthoate biosynthesis II	0.45	2.9E-03
CMP legionaminate biosynthesis I	0.48	1.2E-03
Glycolysis V (Pyrococcus)	0.51	5.0E-04
1, 4 dihydroxy-6-naphthoate biosynthesis I	0.51	3.9E-04

^1^Spearman’s Rho Correlation coefficient.

^2^P-values were adjusted for multiple comparison by false discovery rate.

### Association of Fecal sIgA Levels With Chronological Age and Gut Microbiota Composition

**Figure 6:**
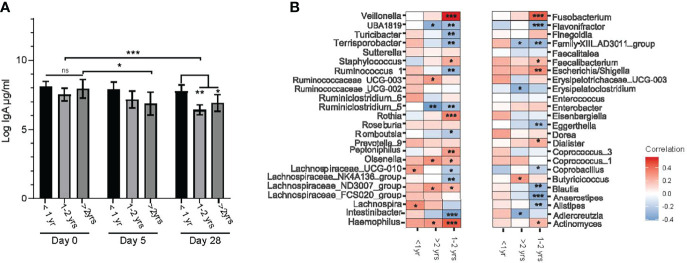
Relationships between the age of study participants, fecal sIgA concentration and genus level bacterial taxa abundance. **(A)** log transformed stool sIgA levels (μg/mL) at entry into the study (day 0) and days 5 and 28 post randomization compared between age categories (under one year of age (<1.0 yr), between one and two years of age (1.0 – 2.0 yrs), and over 2.0 and under 4.0 years of age (>2 yrs). Values are expressed as mean ± SEM. Statistical significance was assessed by using mixed model regression; **(B)** correlation heat map representing Spearman correlation coefficients between changes in center log ratio normalized abundance of bacterial taxa (at the genus level) and log stool IgA levels stratified by age category. Significance is denoted as: *P < 0.05, **P < 0.01 and ***P < 0.001, with multiple comparisons corrected by FDR.

At baseline (day 0) during the acute gastroenteritis episode, there were no differences in sIgA levels among the three age groups. However, significant differences were observed among age groups at both study days 5 and 28 when compared to day 0 (**Figure 6A**). Those >2.0 – <4.0 years of age had a lower sIgA on day 5 than at day 0 (P=0.04). The sIgA levels of the oldest group of participants was also lower than participants 1.0 – 2.0 years of age at the same time point (P=0.03). Secretory IgA levels were unchanged between days 5 and 28 for those <1.0 year of age and those 2.0 – 4.0 years old. To determine whether bacterial diversity impacted sIgA levels in the gut, we performed a mixed model linear regression analysis. Alpha diversity as measured by Shannon index [β=0.322, (-0.170, 0.813) P= 0.2] and Chao1[β= -0.005, (-0.014, 0.004) P= 0.39] did not significantly impact sIgA levels.

We then explored whether there was a relationship between individual bacteria at the genus level and sIgA concentrations by performing correlation analysis between normalized genus level taxa and sIgA within specific age categories. Several bacterial taxa positively correlated with sIgA levels in the 1.0 – 2.0-year-old group, including: opportunistic bacteria *Veillonella*, *Fusobacterium and Haemophilus*. *Blautia, Anaerostipes, Alstipes* and *Intestinbacter* each negatively correlating with sIgA (**Figure 6B**).

## Discussion

These findings demonstrate transient gut colonization of the probiotic strains employed in this prospective randomized trial reported previously (Freedman et al., 2018). The absence of detectable probiotic strains found here at day 28, during the recovery phase of the study, provides evidence of colonization resistance mediated by the commensal colonic microflora. Such transient colonization of the intestine is reassuring that probiotics can be provided for short-term defined interventions without concerns arising from the impact of more protracted changes in the composition and functions of the gut microbiota (Rouanet et al., 2020). Transient gut colonization of preterm infants supplemented with the probiotic *Lactobacillus reuteri*, strain DSM 17938 has been reported, but there was no detectable impact on gut microbial diversity at two years of age (Marti et al., 2021). Of course, it is possible that the transient colonization still could have long-term impacts on the host, including local and systemic immune functions (Vu et al., 2021), which were not evaluated as part of this study. It is also possible that compositional changes in the microbiota of the small intestine was also impacted, but this was not assessed as part of this study due to the relative invasiveness of the methods required to secure small bowel mucosa and luminal fluid for analyses.

We also demonstrate that management of acute gastroenteritis with the probiotic strains *L. helveticus* and *L. rhamnosus* did not result in changes in the composition or deduced functions of the gut microbiome in children 3 to 48 months of age presenting to a hospital emergency department for medical care. The findings indicate that, after accounting for baseline differences between subjects, a 5-day course of treatment with the probiotic mixture of *L. rhamnosus*, strain R0011 and *L. helveticus* R0052 had little impact on the gut microbiota composition. However, a measurable and dominant difference in gut microbiota composition was identified in relation to the age of the child. In addition to confirming several longitudinal cohort studies which also reported an association between bacterial diversity and increased age from infancy to 48 months (Bergstrom et al., 2014; Backhed et al., 2015; Roswall et al., 2021), we detected several linear relationships between changing abundance of microbial functional pathways and increasing chronological age up to 4 years of age. A time-series analysis of intestinal microbiota of 10 children who were between 36 and 48 months of age, had rotavirus-induced acute infectious diarrhea and who received a 5-day course of the yeast probiotic *Saccharomyces boulardii* demonstrated an age-related transition of microbial communities (Dinleyici et al., 2018).

We found no association in gut microbiota composition or microbial diversity to clinical disease severity. However, a decrease in gut microbial diversity was identified in association with the detection of enteric bacterial pathogens; in particular, with the presence of *C. difficile* in stool samples. Asymptomatic carriage of *C. difficile* is common in infancy with several studies finding carriage in up to 80% of infants under 1 year of age (Jangi and Lamont, 2010). While carriage of *C. difficile* is common in infancy, the relationship between *C. difficile* carriage of microbiota diversity is not well characterized in children and infants (Jangi and Lamont, 2010; Rousseau et al., 2011; Drall et al., 2019), with one large cohort study finding significant association between *C. difficile* carriage and feeding modality in infant age 3-4 months. Our results, and those of other researchers (Ling et al., 2014; Hourigan et al., 2019), indicate a relationship between a low complexity community of microbiota and *C. difficile* carriage. It remains to be determined, however, whether the low diversity is a result of *C. difficile* symptomatic carriage (or infection) or a facilitator of bacterial colonization.

Deduced microbial functional differences in the intestinal microbiome also changed with advancing chronological age and altered from the baseline, acute disease, state (day 0) indicating a potential functional association with acute gastroenteritis. During the acute stage of illness, hierarchical clustering analysis revealed that children 1.0 – 2.0 years of age exhibited a more functionally immature gut microbiota, which is more comparable to that of a recovered (day 28) infant <1.0 year. Notably, during the recovery stage (day 28) there were several differential functional pathways associated with participant’s age. Specifically, three functional pathways involving heme biosynthesis all dependant on oxygen and vitamin K biosynthesis were negatively correlated with age. Our observation of a transition from functionally more immature microbiota to a more mature intestinal microbiome parallels the transition from a microaerophilic to more strictly anaerobic gut luminal microenvironment. All three heme biosynthesis pathways require molecular oxygen (Breckau et al., 2003; Frankenberg et al., 2003), indicating that a decline in these pathways correlated with increasing age and could be linked to a transition to a more strictly anaerobic luminal gut microenvironment. As observed previously (Nilsen et al., 2020), we identified a correlation between chronological age and the abundance of metabolic pathways involved in the production of short-chain fatty acids, such as butyrate, by bacteria colonizing the intestinal tract.

This study focused on the composition and deduced functions of bacteria colonizing the gut during the first four years of life. Future studies should capture the role of other microorganisms, such as viruses, bacteriophages, fungi, and protists, as these are also quite likely to have a biological role in human development in both in health and in the context of various disease states (Milani et al., 2018; Rao et al., 2021). While our previous work found no relationship between probiotic administration and secretory fecal IgA levels (Freedman et al., 2021a), within the same cohort we found in the current study relationships between genus level abundance of *Veillonella*, *Blautia* and *Alstipes* and sIgA that were age specific. A previous study focused on the association of immunoglobulins and the infant gut microbiota identified an association between age and declining IgA levels, which was also linked to changes in gut microbiota diversity (Janzon et al., 2019). Herein, we observed a negative correlation between *Blautia* and *Alstipes* and levels of sIgA in stool samples, which is in accordance with the recent finding that *Blautia* evades IgA coating (Janzon et al., 2019) and is associated with a more adult like microbiome composition (Radjabzadeh et al., 2020). These results indicate that a decrease of IgA abundance as well as a rise in the numbers of *Blautia* could provide an indication of increasing maturation of the gut microbiome.

## Conclusion

The findings in this study suggest that for microbiome-based clinical trials, traditional randomization may not be sufficient in capturing individual variations in outcomes, unless substantially large cohorts are utilized. Therefore, studies focused on monitoring the effects of probiotic administration on the composition and the functions of an individual participant’s intestinal microbiome should include both baseline measurements as well as a focus on employing statistics that consider a repeated measure design. In addition to emphasizing the need for including baseline measurements, we also highlight the key subject characteristic of chronological age of the participants is a key factor to be considered when designing future microbiota-based intervention studies during the childhood years.

## Data Availability Statement

The datasets presented in this study can be found in online repositories. The names of the repository/repositories and accession number(s) can be found in the article/**Supplementary Material**.

## Ethics Statement

The studies involving human participants were reviewed and approved by Research ethics boards at each of the six participating Canadian, tertiary-care, university-affiliated sites approved the trial. (clinicaltrials.gov number: NCT01853124). Written informed consent to participate in this study was provided by the participants’ legal guardian/next of kin.

## Author Contributions

RH analyzed the 16sRNA sequence date, developed and analyzed the deduced functional data arising and participated in writing the first draft of this manuscript. SF participated in the conception and design of this study, the acquisition of data and specimens, and interpretation of data. KF, SG, SS, SW-U, NP, KH, and YF participated in the acquisition of data and specimens. X-LP, LC and BL provided the detection and quantification of pathogens in stool specimens. JX contributed to the analysis of data for the work. KJ-H participated in designing this study and reviewed a draft version of the manuscript. LR and MS undertook the 16S rDNA sequencing and reviewed versions of this manuscript. PS participated in the conception of this study and participated in writing the first and subsequent iterations of this manuscript. All authors participate in revising the work for important intellectual content, provided final approval of the version submitted for publication, and agree to be accountable for all aspects of the work.

## Funding

This work was supported by the Canadian Institutes of Health Research (grant nos. 286384 and 325412), a grant from the Alberta Children’s Hospital Foundation to the Pediatric Emergency Medicine Research Associates’ Program, Calgary Laboratory Services (in-kind), the Provincial Laboratory for Public Health–Alberta Public Laboratories, Luminex, and Copan Italia. Study drug and placebo were provided in-kind by Lallemand Health Solutions.

## Conflict of Interest

SF is supported by the Alberta Children’s Hospital Professorship in Child Health and Wellness. MS is the recipient of a Canadian Research Chair in Interdisciplinary Microbiome Research. YF is the recipient of the Canada Research Chair in Pediatric Drug Safety and Efficacy. PS is the recipient of a Canadian Research Chair in Gastrointestinal Disease and research funded by the Canadian Institutes of Health Research (MOP-89894 and IOP-92890) and received honoraria from Abbott Nutrition, Mead Johnson Nutritionals and Nestlé Nutrition.

The remaining authors declare that the research was conducted in the absence of any commercial or financial relationships that could be construed as a potential conflict of interest.

## Publisher’s Note

All claims expressed in this article are solely those of the authors and do not necessarily represent those of their affiliated organizations, or those of the publisher, the editors and the reviewers. Any product that may be evaluated in this article, or claim that may be made by its manufacturer, is not guaranteed or endorsed by the publisher.
