# Supplementary material for: Intestinal Microbial Composition of Children in a Randomized Controlled Trial of Probiotics to Treat Acute Gastroenteritis
Source: Front Cell Infect Microbiol. 2022 Jun 14;12:883163. doi: 10.3389/fcimb.2022.883163 (PMC9238408; doi:10.3389/fcimb.2022.883163)
Supplement: Supplementary file 1 [file DataSheet_1.pdf]

Supplemental Table 1. Clinical characteristics by treatment group.

Variable	Placebo	Probiotic *L. helveticus/ L. rhamnosus*
Day 0 stool sample n (%)	32 (46.4)	37 (53.6)
Day 5 stool sample n (%)	31 (44.9)	38 (55.0)
Day 28 stool sample n (%)	32 (45.7)	38 (54.3)
Baseline MVS score[1] median (IQR)	11 (10- 14)	11 (9.25-13)
Age in months, median (IQR)	15.5 (10-23)	17.5 (12.2-24)
Under 1 year of age n (%)	12 (70.0)	5 (30.0)
1-2 years of age n (%)	12 (35.0)	22 (65.0)
Over 2 years of age n (%)	8 (42.1)	11 (57.8)
Male Sex n (%)	17 (53.1)	19 (0.5)
Received antibiotics in previous 14 d, n (%)	8 (25.0)	4 (10.5))
Pathogen detection n (%)	13 (40.6)	24 (63.1)
Bacterial n (%)	5 (15.6)	6 (15.7)
Virus n (%)	10 (31.3)	18 (47.3)

[1] Scores on the Modified Vesikari Scale score ranges from 0 to 20, with higher scores indicating greater disease severity.
