# Supplementary material for: Intestinal Microbial Composition of Children in a Randomized Controlled Trial of Probiotics to Treat Acute Gastroenteritis
Source: Front Cell Infect Microbiol. 2022 Jun 14;12:883163. doi: 10.3389/fcimb.2022.883163 (PMC9238408; doi:10.3389/fcimb.2022.883163)
Supplement: Supplementary file 2 [file DataSheet_2.pdf]

Supplemental Appendix

Query: NC_018528.1:455041-456601 Lactobacillus helveticus R0052, complete sequence Query ID: lcl|Query

>ASV13
Sequence ID: Query_165898 Length: 429
Range 1: 1 to 429

Score:766 bits(849), Expect:0.0,
Identities:428/429(99%), Gaps:1/429(0%), Strand: Plus/Minus

```
Query  369  TAGGGAATCTTCCACAATGGACGCAAGTCTGATGGAGCAACGCCGCGTGAGTGAAGAAGG  428
            ||||||||||||||||||||||||||||||||||||||||||||||||||||||||||||
Sbjct  429  TAGGGAATCTTCCACAATGGACGCAAGTCTGATGGAGCAACGCCGCGTGAGTGAAGAAGG  370

Query  429  TTTTCGGATCGTAAAGCTCTGTTGTTGGTGAAGAAGGATAGAGGTAGTAACTGGCCTTTA  488
            ||||||||||||||||||||||||||||||||||||||||||||||||||||||||||||
Sbjct  369  TTTTCGGATCGTAAAGCTCTGTTGTTGGTGAAGAAGGATAGAGGTAGTAACTGGCCTTTA  310

Query  489  TTTGACGGTAATCAACCAGAAAGTCACGGCTAACTACGTGCCAGCAGCCGCGGTAATACG  548
            ||||||||||||||||||||||||||||||||||||||||||||||||||||||||||||
Sbjct  309  TTTGACGGTAATCAACCAGAAAGTCACGGCTAACTACGTGCCAGCAGCCGCGGTAATACG  250

Query  549  TAGGTGGCAAGCGTTGTCCGGATTTATTGGGCGTAAAGCGAGCGCAGGCGGAAGAATAAG  608
            ||||||||||||||||||||||||||||||||||||||||||||||||||||||||||||
Sbjct  249  TAGGTGGCAAGCGTTGTCCGGATTTATTGGGCGTAAAGCGAGCGCAGGCGGAAGAATAAG  190

Query  609  TCTGATGTGAAAGCCCTCGGCTTAACCGAGGAATTGCATCGGAAACTG-TTTTCTTGAGT  667
            |||||||||||||||||||||||||||||||||||||||||||||||| |||||||||||
Sbjct  189  TCTGATGTGAAAGCCCTCGGCTTAACCGAGGAATTGCATCGGAAACTGTTTTTCTTGAGT  130

Query  668  GCAGAAGAGGAGAGTGGAACTCCATGTGTAGCGGTGGAATGCGTAGATATATGGAAGAAC  727
            ||||||||||||||||||||||||||||||||||||||||||||||||||||||||||||
Sbjct  129  GCAGAAGAGGAGAGTGGAACTCCATGTGTAGCGGTGGAATGCGTAGATATATGGAAGAAC  70

Query  728  ACCAGTGGCGAAGGCGGCTCTCTGGTCTGCAACTGACGCTGAGGCTCGAAAGCATGGGTA  787
            ||||||||||||||||||||||||||||||||||||||||||||||||||||||||||||
Sbjct  69   ACCAGTGGCGAAGGCGGCTCTCTGGTCTGCAACTGACGCTGAGGCTCGAAAGCATGGGTA  10

Query  788  GCGAACAGG  796
            |||||||||
Sbjct  9    GCGAACAGG  1
```

_165884 Length: 1561
